# Sustainable metabolic engineering for sustainability optimisation of industrial biotechnology

**DOI:** 10.1016/j.csbj.2021.08.034

**Published:** 2021-08-25

**Authors:** Egils Stalidzans, Elina Dace

**Affiliations:** Institute of Microbiology and Biotechnology, University of Latvia, 1 Jelgavas Street, Riga LV1004, Latvia

**Keywords:** Biotechnology, Genome-scale metabolic models, Mathematical modelling, Ranking, Sustainability optimisation, Sustainable metabolic engineering

## Abstract

Industrial biotechnology represents one of the most innovating and labour-productive industries with an estimated stable economic growth, thus giving space for improvement of the existing and setting up new value chains. In addition, biotechnology has clear environmental advantages over the chemical industry. Still, biotechnology’s environmental contribution is sometimes valued with controversy and societal aspects are frequently ignored. Environmental, economic and societal sustainability of various bioprocesses becomes increasingly important due to the growing understanding about complex and interlinked consequences of different human activities. Neglecting the sustainability issues in the development process of novel solutions may lead to sub-optimal biotechnological production, causing adverse environmental and societal problems proportional to the production volumes.

In the paper, sustainable metabolic engineering (SME) concept is proposed to assess and optimize the sustainability of biotechnological production that can be derived from the features of metabolism of the exploited organism. The SME concept is optimization of metabolism where economic, environmental and societal sustainability parameters of all incoming and outgoing fluxes and produced biomass of the applied organisms are considered. The extension of characterising features of strains designed by metabolic engineering methods with sustainability estimation enables *ab initio* improvement of the biotechnological production design.

## 1 Introduction

Industrial biotechnology has changed the way chemicals, energy, materials, food, and other products are produced. Biotechnology has several clear environmental advantages over the chemical industry as (1) the use of renewable bioresources instead of non-renewable petroleum-based resources; (2) production of biodegradable products instead of nondegradable and/or non-recyclable products; (3) avoidance of toxic industrial waste and gases, etc. Still, biotechnology’s environmental contribution is sometimes valued with controversy. Biotechnology uses biomass as the fermentation feedstock presuming its renewability and carbon-neutrality. Yet, resource inputs required to obtain and process the biomass feedstock, especially in its pure form (e.g., glucose), for its use in the biotechnological production process should be considered to assess the total environmental load. An additional point of discussion is the competing use of land for growing biomass for fuel or chemical feedstock production versus growing foodstuffs and maintaining biodiverse forests that serve as the global carbon sink.

The growing biotechnology represents one of the most innovating and labour-productive industries of the bioeconomy sector by value added. Bioeconomy has gained traction as a driver towards smart and green growth. More than 40 nations are currently investing and promoting strategies and policies in the bioeconomy sector. In 2017, the European Union’s (EU) bioeconomy sector employed more than 417 thousand people in the manufacture of bio-based chemicals, pharmaceuticals, plastics, rubber, and liquid biofuels, which generated 63,528 million euro of value-added (about 10% of the bioeconomy sectors’ total value-added) [1]. With bioeconomy, circular economy, sustainable development, and climate neutrality strategies high on the international agenda creating ‘green jobs’ and revenue based on innovative biotechnological solutions are expected to be further actively promoted. This agenda means that biotechnological solutions will grow not only in numbers but also in scale. Neglecting the environmental, economic, and societal issues in the development process of novel solutions may lead to sub-optimal biotechnological production, causing adverse environmental and societal problems proportional to the production volumes. It is anticipated that in the near future, a detailed economic, environmental and societal sustainability assessment and optimisation of a biotechnological process will become standard for the industry due to the increasing societal and governmental pressure to lower environmental impact on ecosystems, human health, and resource availability [2].

The prominent presence of the UN Sustainable Development Goals [2] prioritizes research directions needed for the sustainable future. Seventeen interconnected goals cover agriculture, climate change, industry, innovations, and other sectors, and bioindustry has a role to play [3]. Nevertheless, sustainability remains a disputable topic, even when researchers agree on one common definition of sustainability. Various aspects of sustainability can be evaluated differently [4], and depend on the aim, boundaries, and stakeholder involvement in the evaluation. Among stakeholders, this creates a lack of shared understanding of how sustainability can be achieved and the sustainable development goals operationalized [5]. In the context of industrial biotechnology, we may define sustainability as optimized economic, environmental and societal costs and benefits of production by efficient utilization of renewable resources, elimination of gaseous, liquid or solid waste generation, and avoidance of toxic substances by ensuring biosafety and biosecurity, compliance with ethical standards, and acceptance by society to achieve profitable production and processing of bio-based products.

The sustainability of different bioprocesses becomes increasingly important parameter due to the growing understanding about consequences of different human activities [6], [7], [8]. The intention of EU and other regions is to develop stimulating mechanisms to facilitate implementation of industrial sustainability. That is the point where sustainability shifts business models and impacts profit along with classic parameters of bioprocess as yield and productivity and others making the assessment of competitiveness of a particular solution even more complicated.

Mathematical modelling and assessment are the only option to assess the impact of current technological limitations on the potential of improvements (optimal solution utilising available technologies) without having high number of ‘wet’ experiments. Modelling can describe the whole solution space constrained by the current technological alternatives and search optimum within the huge space of alternatives with different optimisation methods and algorithms. Modelling can be expensive, but it may turn out relatively cheap comparing to a potential failure of investments. We acknowledge that there are many processes where modelling cannot help much, for instance, due to our limited understanding about the functionality of organisms under different conditions. Still, even when models can not describe all details, it is wise to assess the optimisation potential early at the bioprocess design workflow to use modelling for the elements and aspects where methods and tools are available. The known reliable constrains (e.g., mass and energy conservation and others) may provide enough information to find the optimum assuming optimistically that unknown details do not reduce the attractivity of solution to an uninteresting level. If the changes are not attractive even with the optimistic assumptions, the question is answered – the proposed solution is not worth implementing.

The metabolism of an organism exploited in a biotechnological process determines many features of the. Therefore, metabolism at cellular level is a popular optimisation target addressed by metabolic engineering – purposeful modification of metabolic networks of biochemical reactions [9]. The branch of metabolic engineering emerged as a rational approach to change metabolism with two steps – *analysis* and *synthesis* – just as in all other fields of engineering [10]. Mathematical models are used for the analysis: optimisation of metabolic network and the assessment of the consequences of potential changes before the implementation of organism “design”. Metabolic engineering is also one of synthetic biology’s tools [11]. Still, in many cases the engineering ideas are generated intuitively keeping in mind a specific organism and product, and the engineering process is built around that idea. To find the full scope of optimisation potential offered by available technologies, a model-based screening of solution space and ranking of designs according to the selected criteria is necessary [12].

The aim of the paper is to discuss the application, options and limitations of mathematical modelling of metabolism in facilitating the sustainability of industrial biotechnology by introducing the sustainable metabolic engineering concept.

## 2 Assessment of sustainability of biotechnological production

**Fig. 1:**
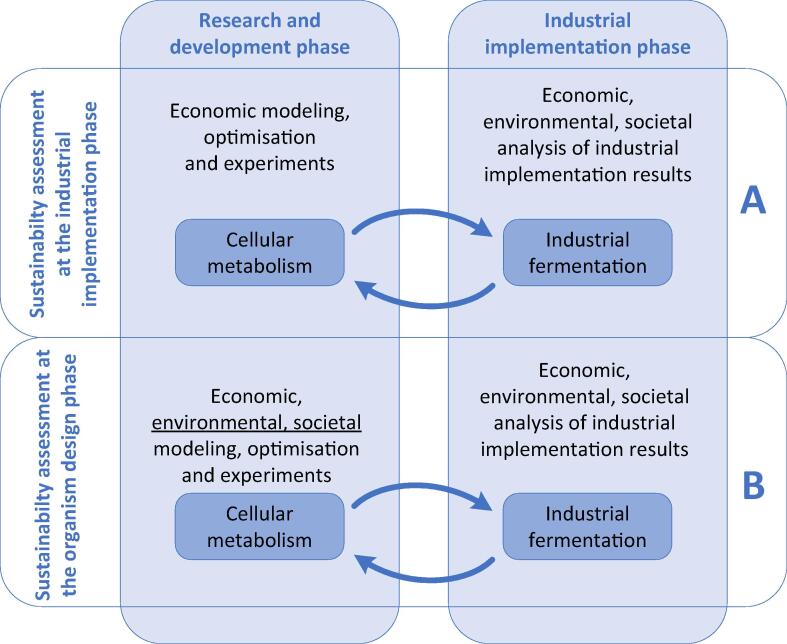
Implementation of sustainability analysis at the design (A) and implementation (B) phases of biotechnological production.

A typical sequence of activities setting up a biotechnological production is the selection of economically beneficial product and/or substrate pair or set. Then appropriate organism or modified strains for the production are selected. It may as well work in the opposite direction when appropriate target products are sought for an organism with specific features to exploit them. Nonetheless, once the organism and product or substrate are selected, many features of the biotechnological production become determined and the rest of potentially influencing parameters have to be accepted as given [8] (A in Fig. 1). A feedback from the industrial implementation phase on the changes needed in the metabolism may lead to re-selection of the organism or its modifications that echo in costs and time to correct the industrial implementation.

An alternative is to invest more in the modelling at the design phase (B in Fig. 1) expanding the analysis of the model-based optimisation with even higher complexity by including parameters that characterize sustainability. That leads to a more detailed and, therefore, reliable evaluation of the potential strategies. Introducing the economic, environmental, and societal criteria at the phase of selection of the substrate, product and/or modifications made to an organism (B in Fig. 1) enables a comprehensive analysis and optimisation at the mathematical modelling phase. The task is complex, as each metabolic flux of media components or by-products that enters or leaves the organism contributes not only to economic (buying or selling), but also to the environmental (e.g., efficient use of resources, emissions to air, soil, and water), and societal impacts (number of employed persons, societal attitude to the producing organism, media compounds or by-products) of the industrial biotechnology. Complex analysis is necessary to take into account potential conflicts between the three sustainability components. Genetic modifications, that are useful from the engineering perspective may be disadvantage in the social perception. Meanwhile, an increase of yield may create negative environmental impacts due to changes in the related by-product spectrum.

**Fig. 2:**
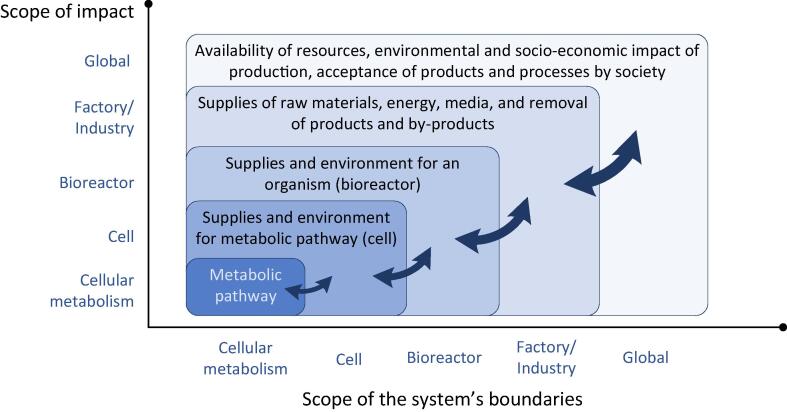
The scope of impact vs. the scope of system’s boundaries in biotechnological production.

An intensive modelling implementation at the research and development phase (B in Fig. 1) demands a decision about the system’s boundaries (Fig. 2). The wider is the modelling scope the more the sustainability aspects must be considered. Meanwhile, the accuracy of predictions decreases. Still, there are some aspects (energy, mass) that can be calculated at high accuracy and “it is better to be roughly right than exactly wrong” as said by John Maynard Keynes. And modelling is a mean to be “roughly right” even in case of information insufficiency.

Looking at the exchange between the system’s scales (Fig. 2), parameters determining the sustainability of a biotechnological production start at the exploited metabolic pathway of the producing organism. The producing metabolic pathway (or set of pathways) connects substrates with products at some yield and productivity. The pathway operation must be secured by metabolites, cofactors, enzymes, and other elements at the cellular metabolism level by other metabolic pathways that also consume some compounds of media and produce by-products, hence determining operation costs, environmental impact, and societal acceptance. Thus, it is reasonable to start the optimisation of sustainability of production at the level of organism that contains the specific pathways.

At the bioreactor and factory/industry scale the techno-economic analysis (TEA) [13] can be applied searching for the best technological alternatives of bioprocess implementation at technological readiness levels (TRL) 5 and higher. While life cycle assessment (LCA) [14] can be used at factory/industry scale at TRL 7 and higher.

## 3 Sustainable metabolic engineering

Metabolic engineering deals with the complexity of interlinked pathways up to genome scale and has the potential to significantly increase the amount, plurality and variety of products obtained from renewable feedstocks [8]. The importance of sustainability in industrial biotechnology has been recognized in several studies before, yet mostly limited to the biogenous origin of the feedstock used or product produced [8], [15]. In many cases, research has included only environmental (by e.g. LCA) or economic (by e.g. TEA, cost-benefit analysis) assessment ignoring or unable to capture social impacts of the biotechnology. In addition, such assessment studies most often focus on comparative assessment of the proposed bio-based solution versus conventional products [16], or their production processes [17]. Meanwhile, Straathof et al. [18] note that a full sustainability analysis should be performed during the design phase referring to the whole biotechnology supply chain.

For biotechnological processes to be truly sustainable, a set of factors must be considered right at the beginning of the research and development phase. There has been an attempt to create a framework for assessing the economic and environmental performance of a biochemical production formulated as a metabolic model [19]. Yet inclusion of the sustainability indicators already in the design phase of a biotechnological microorganism has not been attempted before. We propose to assess the sustainability features of a biotechnological process *ab initio* – during the selection of the organism and its optional modification (engineering) as the consequences of organism selection dictate most of other interactions (Fig. 2). Therefore, the selection of producing metabolism executed by selected organism must be optimized or at least assessed in all sustainability aspects – economic, environmental, and societal. That would reduce the possibility of unexpected outcomes at the industrial implementation phase (Fig. 1). Fortunately, one of the scientific disciplines where mathematical modelling of biochemical processes can make rather good predictions, is the metabolism, as metabolites are relatively easy to measure and even more important – the mass conservation law enables implementation of several rules (mass balance of reactions) and assumptions (steady state) [20].

**Fig. 3:**
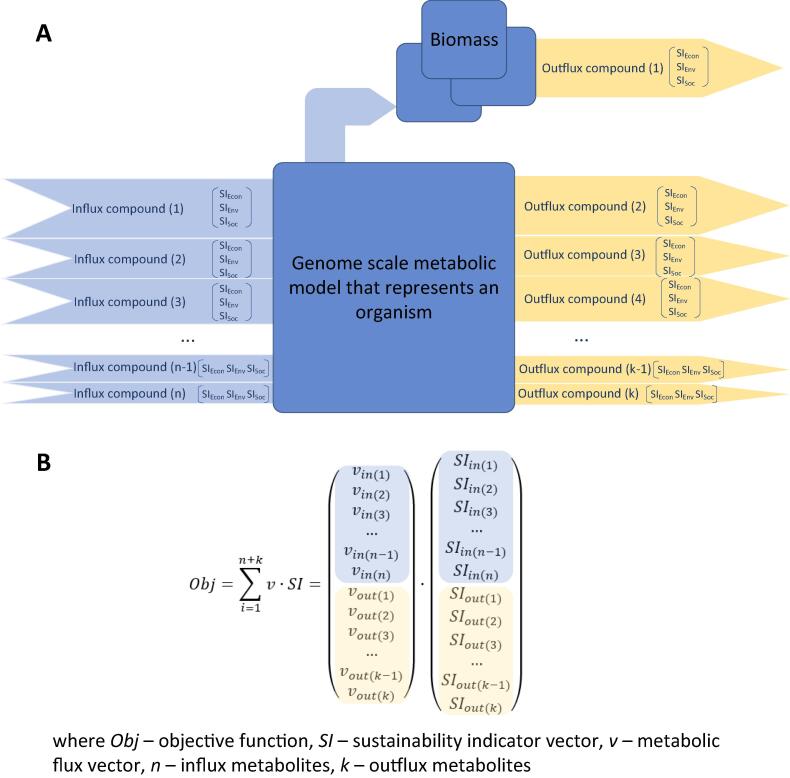
Formation of sustainability indicator score by metabolic influxes and outfluxes (A) and definition of the objective function with inclusion of the sustainability indicators (B).

Among different modelling approaches, constraint based stoichiometric modelling [21], [22] stands out using genome-scale metabolic models (GSMMs). They can capture the whole metabolism of an organism providing a system of mass balanced reactions that can account for every atom, electron and proton that enters or leaves the system [23]. That means – all influxes and outfluxes of the organism can be predicted at some accuracy if the metabolic flux variability is narrowed during metabolic engineering [24]. The full overview of exchange of metabolites between the cells of an organism and a bioreactor (Fig. 2) can be accounted by estimating costs and benefits and mass exchanges at the interconnected scales of the system. Thus, at the phase of metabolic engineering it is possible to include economic, environmental, and societal parameters that *ab initio* provides sustainable outcome for the industrial implementation phase. It means that the sustainability criteria are included already in the selection and design of an organism. Metabolic engineering can be coupled with sustainability analysis by incorporating sustainability indicator score formed by the impact of individual metabolic exchange fluxes (Fig. 3A) in the objective function of metabolism optimization. The extension of the scope of an objective function presents a new meaning to the metabolic engineering and leads to an early-stage (*ab initio*) sustainability assessment and inclusion of its diverse aspects that in detail have not been considered before.

We propose the **Sustainable Metabolic Engineering (SME)** concept – optimization of metabolism where economic, environmental and societal sustainability parameters of all incoming and outgoing fluxes and produced biomass of the applied organisms are considered.

GSMM optimization by constraint-based stoichiometric modelling, applied in metabolic engineering context, currently seems to be the most appropriate way to gain an overview about the expected exchange of metabolites between the organism and the environment. To implement the SME concept, the objective function of the optimisation process must include more than typical biomass, product flux and/or yield maximisation. The value of the objective function should be calculated by multiplying the vector of metabolite exchange fluxes obtained from genome-scale stoichiometric metabolic models by the corresponding vector of sustainability parameters that characterize the metabolite exchange fluxes (Fig. 3B). The SME concept is supported by automated GSMM building software products bringing more than 6000 automatically or manually built GSMMs [25]. There is also a number of freely available optimisation software tools where the sustainability vector can be introduced: *COBRA v3.0* [26], *Raven* [27] *Merlin* [28] and others [29] available for this purpose.

According to the SME concept, optimisation and ranking of metabolic engineering designs is executed by including economic, environmental, and societal parameters in the objective function determined by the balance of metabolic flux exchange. The economic parameters that can potentially be included in the sustainability calculation of the metabolic flux set are, for example, media compound costs, market price of product components, and waste management costs (or negative price of the produced components) [30], [31]. Environmental impacts can be assessed according to the well-established LCA methodology [32] and media compounds and product components can be characterized by their aquatic acidification potential, aquatic eutrophication potential, aquatic ecotoxicity potential, terrestrial eco-toxicity potential, terrestrial acidification/nutrification potential, abiotic depletion potential, and global warming potential [33], [34] calculated based on the data available in such databases as *Ecoinvent* or *GaBi* and others [35]. The LCA impact categories or other relevant approaches [36] can be used to also assess the societal sustainability via e.g., the human health impacts of the media compounds and product components by their human toxicity potential, ozone depletion potential, photochemical oxidant creation potential, as well as impacts of urban, agricultural, natural land occupation potential, public opinion and social acceptance, and jobs created. Thus, the necessary elements for *ab initio* sustainability assessment are available and must be applied in a coordinated manner with the metabolic exchange obtained by metabolic modelling.

It is important that the objective function that is the exchange flux vector multiplied by the sustainability vector associated with economic, environmental and social criteria (Fig. 3B), enables automatic optimisation by the existing software tools after some modifications. That means applicability of machine learning and other approaches [37] that help to deal with the large solution space offered by the available GSMMs and metabolic reaction databases.

The implementation of SME concept enables detailed analysis of the spectrum of organisms, media compounds and produced components due to their impact on the objective function as (1) the most appropriate organism can be selected as chassis, (2) the spectrum of product components might be metabolically engineered to avoid metabolites characterized by negative sustainability impact, (3) media compounds might be replaced by more sustainable compounds. As a result, metabolic engineering can be applied to handle even higher complexity of optimisation aroused by the sustainability goal included in the objective function.

The optimisation results (organism designs) can be ranked according to the sustainability indicator score where each design is characterised by the selected chassis organism, number of reaction deletions and insertions and weighted sum of economic, environmental and social score compounds for a particular substrate/product set.

An important issue in the implementation of the SME concept is the flux variability [38]. The sustainability assessment should be done considering the flux variability – the range of flux values of specific reactions that ensure the optimum metabolic result. To make the model predictions of sustainability meaningful, flux variability must be reduced by selection of appropriate media or introduction or deletion of reactions until the variability of the critical fluxes, to the least, have an acceptable flux variability in respect to sustainability. Flux variability reducing metabolic engineering has already been performed for several products before [24], [39].

All metabolic exchange fluxes mentioned in the definition do not necessarily mean that sustainability parameter values must be found for each metabolite. The impact of small fluxes below some threshold may be neglected or lumped, yet that becomes possible only after their values are calculated as a result of genome-scale metabolic modelling. On the other hand, some metabolites may have high impact on the sustainability even when the flux is relatively small. The sustainability indicator vector elements can contain also different functions to take into account non-linearity. Meanwhile, it may be complicated to consider the non-linear impacts between various sustainability goals (see Section 1). Social assessment can be especially challenging, as there may be insufficient data on specific emerging bioprocesses requesting for additional research and data collection.

The constraint based stoichiometric modelling at genome-scale can be combined with other methods (e.g. LCA, life cycle sustainability assessment, TEA, environmental footprint, eco-design and others) to improve the accuracy of sustainability assessment and the biotechnological feasibility of the engineered strain. The kinetic modelling can be used to approach the main production pathways with higher accuracy considering also the impact of metabolite concentration on the throughput of the pathway [40]. Important addition to the metabolic engineering approaches is the resource allocation modelling [41] and other constraints [20] to increase the feasibility of organism designs.

## 4 Summary and outlook

The sustainability criterion of industrial biotechnology should not be limited to the use of renewable resources or production of bio-based products. Sustainability of industrial biotechnology must also incorporate the zero-waste and zero-emission concepts, biosafety, biosecurity and social acceptance of the process and products along with overall economic benefits. It is technically feasible to assess the optimality of a biotechnological production in all aspects of sustainability – economic, environmental, and societal. The set of all fluxes consumed and produced by an organism provides enough information for the sustainability assessment. A full balance of fluxes entering and leaving the organism can be calculated and optimised by the well-developed constraint based stoichiometric genome-scale metabolic modelling and optimisation. The extension of metabolic engineering with sustainability optimisation functionality (sustainable metabolic engineering) provides a whole new dimension in the design of the biotechnological production.

## CRediT authorship contribution statement

**Egils Stalidzans:** Conceptualization, Methodology, Visualization, Writing – original draft, Writing - review & editing. **Elina Dace:** Conceptualization, Methodology, Visualization, Writing – original draft, Writing - review & editing, Project administration.

## Declaration of Competing Interest

The authors declare that they have no known competing financial interests or personal relationships that could have appeared to influence the work reported in this paper.
